# Death of Dr. Isaac Parrish

**Published:** 1852-09

**Authors:** 


THE MEDICAL EXAMINER.

PHILADELPHIA, SEPTEMBER, 1852.

### OBITUARY.

We regret to announce the death of Dr. Isaac Parrish, of this city, by dysentery, after a short illness.

Few men could be less spared than Dr. Parrish; he was essentially a working man in the profession, both in the daily routine of practical duties and in everything calculated to advance and elevate it. To the pages of this journal, as well as of many of the other medical and daily presses, he was a constant and welcome contributor, and his articles were always characterised by sound judgment and practical bearing. He died in the prime of life and in the full tide of a successful practice, beloved and respected by all who knew him.

